# Effects of Maximal Strength Training on Perceived-Fatigue and Functional Mobility in Persons with Relapsing-Remitting Multiple Sclerosis

**DOI:** 10.3390/medicina56120718

**Published:** 2020-12-20

**Authors:** Ramon Gomez-Illan, Raul Reina, David Barbado, Rafael Sabido, Pedro Moreno-Navarro, Alba Roldan

**Affiliations:** Sport Research Centre, Department of Sport Sciences, Miguel Hernandez University, 03202 Elche, Spain; rjgi78@gmail.com (R.G.-I.); dbarbado@umh.es (D.B.); rsabido@umh.es (R.S.); p.moreno@umh.es (P.M.-N.); aroldan@umh.es (A.R.)

**Keywords:** maximal strength training, Fatigue Severity Scale, isokinetic strength, isometric strength, quality of life, lower limbs

## Abstract

*Background and objectives*: Fatigue is one of the most disabling symptoms that limit daily life activities in persons with multiple sclerosis (pwMS). This study aimed to evaluate the effects of maximal strength training (MST) on perceived-fatigue and functional mobility in pwMS. *Materials and Methods*: 26 participants with MS were balanced according to their pre-intervention fatigue scores and distributed into an MST group (*n* = 13) and a control group (CG; *n* = 13). The MST group completed eight weeks using high loads, evaluating detraining after ten weeks. Quadriceps and hamstring isokinetic (QPT_IK_; HPT_IK_) and isometric (QPT_IM_; HPT_IM_) peak torques were assessed using an isokinetic dynamometer. Effect size differences were estimated with the Hedges’ *g* index (*d_g_*). Fatigue was evaluated through the Fatigue Severity Scale (FSS), while functional mobility was assessed via the Timed Up and Go Test (TUG). *Results*: The MST significantly improved all the strength measurements after the intervention (Δ6.43–29.55%; *p* < 0.05) compared to the control group. FSS showed a significant reduction (59.97%, *d_g_* = 5.41, large). The MST group also reduced the TUG time (19.69%; *d_g_* = 0.93, large) compared to the control group. Improvements caused by the intervention did not remain after a 10-week follow-up, with decreases in strength performance from 4.40% to 13.86% (*d_g_* = 0.24–0.56, small to moderate), 112.08% in the FSS (*d_g_* = −3.88, large), and 16.93% in TUG (*d_g_* = −1.07, large). *Conclusions*: MST (up to 90% 1RM) seems to be a feasible and useful way to obtain clinically relevant improvements in the perceived-fatigue symptoms and functional mobility. Still, symptom improvements decrease after a 10-week detraining period.

## 1. Introduction

Fatigue has been identified as one of the most important and frequent symptoms that negatively affect the quality of life (QoL) of people with multiple sclerosis (pwMS) [1]. Usually, three of every four pwMS indicate experiencing symptoms of fatigue at least once per week [2], which is one of the main causes of unemployment [1]. Besides, high levels of fatigue are usually accompanied by other symptoms such as depression, pain, anxiety or cognitive dysfunction [3], and also functional mobility reduction, which will determine the participation of pwMS in the community [4]. Despite the impact that fatigue has on the QoL, the exact causes of fatigue in MS have not been determined. Fatigue seems to be related to the typical neurodegeneration process of the pathology itself (central fatigue) and physical inactivity [5]. Among the potential therapies to reduce the fatigue symptoms in MS, several researchers have confirmed that physical training programs can be a safe [6] and effective tool [5,7] to reduce the fatigue at the same time that improves other symptoms like as balance impairments or strength deficits [8].

There is no clear consensus on the type of training programs that could achieve a significant reduction in fatigue in pwMS. The current empirical evidence does not consider any one particular training method as better than any other [7]. Nevertheless, resistance training appears to have some advantages above other exercise regimens [9] as it produces improvements in neural drive [10] as well as in the efferent motor output of spinal motor neurons in pwMS [11]. This leads to enhancements not only in strength but also in balance [11] and functional mobility [12,13], which are particularly important for the QoL of this population [11]. Additionally, resistance training seems to be better tolerated by this population (especially by the most sedentary individuals), as their body temperature does not increase overmuch. The increase in temperature in pwMS is related to loss of physical performance and low states of mood [14].

Despite the benefits that resistance training programs can trigger in pwMS’ general physical condition and QoL, they have not always delivered as positive results as could be expected for reducing fatigue in pwMS [7]. Although resistance training programs have been postulated as an adequate tool to reduce fatigue, the effect size (ES) observed in the majority of studies are low or even trivial (0.10 < ES < 0.48) [7]. A possible reason behind the limited positive effects of resistance training on fatigue may be that most studies have conducted progressive programs up-to-sub-maximum loads (<80% of 1 repetition maximum -RM-) [15]. However, it is known that maximal strength training (MST), which uses loads higher than 80% 1RM, requires the complete use of the neuromuscular system, enhancing the recruitment of muscle fibres and neural drive in a higher extent than other resistance training [16]. Based on this feature, MST seems to be an adequate resistance training regimen to cope with the decreased central neural drive associated with MS, which, in turn, could ameliorate some of the disease symptoms. Accordingly, MST (≥80% of 1RM) on this population has also been shown to be a useful methodology to reduce peripheral pro-inflammatory cytokine levels [17], which seems related to fatigue symptoms during the disease [18]. Based on these preliminary findings, high training loads could be a key component of resistance training to reduce fatigue in pwMS. However, a pilot study applying high loads (85–95% of 1RM) did not find a significant reduction in perceived-fatigue after eight weeks of intervention, questioning the effectiveness of MST in reducing this symptom [19].

To clarify the current controversy, this study aimed to analyse the effect of an MST program on perceived-fatigue in pwMS. Additionally, since functional mobility improvements are considered an important factor in reducing fatigue caused by daily life physical activities [19], the potential benefits of the MST on this parameter were also explored. We hypothesized that: (1) the MST program will lead to a reduction in perceived-fatigue in pwMS compared to a control group, and (2) the MST program will improve functional mobility compared to a control group.

## 2. Materials and Methods

### 2.1. Participants

A convenience sample of twenty-six participants (43.73 ± 10.12 years old; Expanded Disability Status Scale (EDSS) = 2.58 ± 1.19), were selected from patients of the Neurology Department of a Spanish Public Hospital, participating voluntarily in the study if they complied with the following inclusion criteria: (1) to be a patient with relapsing-remitting MS diagnosed by a neurologist, (2) to have symptoms of severe perceived-fatigue (>36 points on the Fatigue Severity Scale (FSS)), (3) to possess <6.5 points on the EDSS, and (4) to have availability for attending the whole training and testing sessions. The head physician of the Neurology Department agreed with the participation of their patients in the program, and the study was approved by the University’s Ethics Committee where the research took place (reference number DPS.RRV.02.14, approved on 25 January 2015). All participants signed an informed consent for their involvement in the study. Patients were randomly and balanced assigned into two groups: the intervention group, which performed the MST program, and the control group (CG), which did not perform any regular physical exercise during the period where the study was conducted.

### 2.2. Experimental Procedures

#### 2.2.1. Isokinetic and Isometric Strength Measurements

An isokinetic dynamometer (Biodex System 4 PRO, Biodex Medical Systems, Shirley, NY, USA) was used to evaluate lower limb isokinetic and isometric strength, both knee extensors (i.e., quadriceps) and flexors (i.e., hamstrings). The dynamometer seat was adjusted to each participant, strapping chest, waist, and the involved leg to isolate the joint action and impeding the movement of the rest of the body during testing. The dynamometer torque was positioned 2 cm from the axis of the knee and the leg of the involved joint was held together by the torque, using the articulated arm provided by the manufacturer. Participants’ arms remained crossed over the chest throughout the test, and the data regarding the adjustments were recorded for the following test sessions of measurement. Before each test, participants underwent a 5 min warm-up on a stationary bicycle.

**Figure 1:**
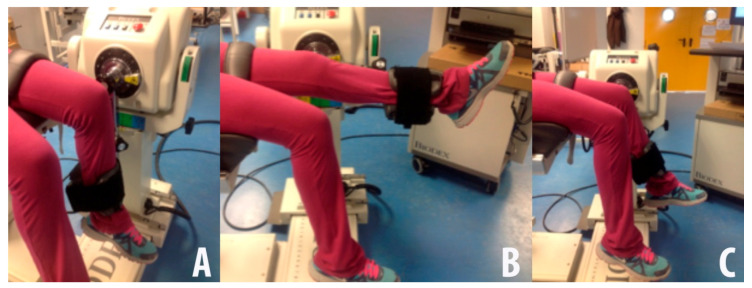
Isokinetic protocol, with start (maximal flexion: 90°) (**A**) and final (maximal extension: 10°) (**B**) positions; Isometric protocol (70° position) (**C**).

For the isokinetic evaluation, participants started from an anatomical position of the knee bent at 90°. Participants carried out two sets of five repetitions of knee extension/flexion at a speed of 60°/s in a range of movement of 80° (from 90° to 170°). Participants rested 3 min between sets. The Isokinetic Peak Torque (PTIK) was taken to be the maximum value in Newton × meter (N × m) reached, both in extension and in flexion in any of the two sets (Figure 1A,B).

For the isometric protocol, starting from an anatomical reference position with the knee bent at 90°, the arm supplied by the manufacturer was fixed at 70°. The subjects carried out three sets of voluntary maximum contractions of the quadriceps (attempt of extension) for 5 s, followed by another three sets of the hamstrings (attempt of flexion) for 5 s, with 15 s rest between contractions. The rest period between sets was 60 s. The Isometric Peak Torque (PTIM) was taken to be the maximum value in Newton × meter (N × m) reached, both in extension and in flexion in either of the three sets (Figure 1C). To simplify the subsequent statistical analyses of the strength variables (PTIK and PTIM) results from the left and right legs were averaged and normalized by the bodyweight [(right leg + left leg)/body mass] [20].

#### 2.2.2. Perceived-Fatigue

The FSS [21] was used to quantify pwMS’ perceived-fatigue in weekly periods, asking about nine situations in which the participant responds on a 1-to-7 Likert scale, where 1 means ‘strongly disagree’ and 7 means ‘strongly agree’ (min = 9 points, max = 63 points). This scale has shown good reliability, with Cronbach’s alpha of 0.88 and 0.81, respectively. Greater FSS scores mean greater perceived-fatigue.

#### 2.2.3. Evaluation of Functional Mobility

The Timed Up and Go Test (TUG) was used to evaluate the participants’ functional mobility [22]. TUG has good inter-rater and intra-rater reliability (ICC = 0.99) in pwMS [23]. Participants performed three repetitions with 1 min rest between trials, and the average of the two best trials was used for analysis.

#### 2.2.4. Intervention

Data collection was carried out at a university’s sports research centre lab, and nearby sports facilities. Facilities temperature was set up at 23 °C and participants were required not to exercise 48 h before the evaluation sessions. Participants performed three testing sessions: pre-test, post-test (8-weeks after pre-test), and follow-up test to evaluate detraining (10-weeks after the end of the intervention). In these three testing stages, participants performed, in this order, the perception scales of fatigue (FSS), the isokinetic and isometric strength tests, and the TUG. All the testing and training sessions were conducted by the same researchers, with PhD and/or master’s degree in sports sciences.

Before the MST program, participants underwent a four-week conditioning period, three training sessions per week, based on endurance strength exercises with the objective of guarantee the correct performance of the strength exercises, the familiarization with the gym machines and work routines. After this period, the eight-weeks MST program was carried out continuing with the three training sessions scheduled per week. The first two weeks of the MST program were used for the transition to the high-intensity loads, and after the third week, the training with high-loads was fully implemented until the end of the intervention period (i.e., a total of six weeks) (Table 1). The load progression carried out in this study was based on previous works applying resistance training in people with multiple sclerosis [24,25].

During this intervention, none of the participants undertook any parallel physical activity to the study. Two days before the beginning of the intervention period, one-Repetition Maximum (1RM) test was carried out employing the Brzycki protocol [26,27] to individualize training loads for each resistance exercise, expressed as a percentage of that 1RM. During the follow-up, participants were encouraged to keep the daily-life routine that they used to do before the study intervention.

All sessions had the same structure, beginning with 5 min of cardiovascular exercises (treadmill, static bicycle, or walking) followed by the program designed for each day. Stretching was carried out on the worked muscle groups just after each exercise and at the end of the sessions as a cool down. Sets, repetitions, rest intervals, and %1RM are also described in Table 1. During the conditioning period, all the participants carried out the same exercises each day. During the intervention period, participants were divided into three working groups, where each group performed one of the three weekly workouts each day (Table 2). Every week, the resting time was 24 h between sessions one and two, and 48 h between sessions two and three. No adverse events occurred during the training period and data collection.

### 2.3. Statistical Analysis

Descriptive statistics (mean and standard deviation) were used to present data. All the variables showed a normal distribution according to the Kolmogorov-Smirnov test with the Lilliefors correction. ANOVAs for repeated measures were performed for all variables to test differences between groups, being intervention the between-group factor (2 levels: MST, CG) and moment of evaluation the within-group factor (3 levels: pre-test, post-test, and follow-up test). Two effect size indexes were used to assess the practical signification within and between-group differences. On one hand, Partial eta-square (ηp^2^) values were calculated as a measure of effect size for mean differences in the repeated-measures analyses with the following interpretation: above 0.26, between 0.26 and 0.02, and lower than 0.02 were considered as large, medium, and small, respectively [28]. On the other hand, Hedges’ *g* effect size index (*d_g_*) [29] was calculated to assess the practical signification of within and between-group differences. This index is based on Cohen’s d index [30] but it provides an effect size estimation reducing the bias caused by small samples (*n* < 20), interpreted as follows: large (*d_g_* > 0.8), moderate (0.5 < *d_g_* < 0.8), small (0.2 < *d_g_* < 0.5) and trivial (*d_g_* < 0.2). Besides, to provide more clinically meaningful information about the training effects, percentages of improvement (%) of each variable were also calculated as follows: intervention improvement (pre-test vs. post-test) and detraining (re-test vs. follow-up test). The statistical analysis was conducted with the Statistical Package for Social Sciences (version 22.0, SPSS Inc., Chicago, IL, USA), with the significance level chosen at *p* < 0.05.

## 3. Results

Table 3 shows the sample demographics considering age, body mass, perceived fatigue and functional proficiency measured by the TUG test. No significant differences were found between groups when comparing the pre-intervention scores after the random allocation of the participants to the MST and CG, respectively.

The repeated-measures ANOVA revealed interactive effects between the within and the between group factors in all the strength variables: QPT_IK_ [*F*(1,24) = 41.71; *p* < 0.001; ŋρ^2^ = 0.64], HPT_IK_ [*F*(1,24) = 12.94; *p* < 0.001; ŋρ^2^ = 0.35], QPT_IM_ [*F*(1,24) = 22.84; *p* < 0.001; ŋρ^2^ = 0.49], and HPT_IM_ [*F*(1,24) = 6.84; *p* = 0.015; ŋρ^2^ = 0.22]. Significant differences were also obtained for the FSS scale [*F*(1,24) = 87.85; *p* < 0.001; ŋρ^2^ = 0.78], while a moderate effect size was obtained for the TUG test [*F*(1,24) = 3.38; *p* = 0.079; ŋρ^2^ = 0.12].

Table 4 shows the repeated-measures analyses for the MST group and the CG, including pairwise comparisons. When comparing pre- vs post-intervention assessments, the MST group significantly improved their knee extension and flexion strength scores in all the isokinetic and isometric exertions compared to the CG (Δ6.43−29.55%; *p* < 0.05). However, this improvement is only maintained for the knee flexion strength (Δ22.73%; *d_g_* = −0.78, moderate) when comparing the pre-test scores with the follow-up measurements. The post- vs follow-up test comparison revealed significant decreases of the isokinetic and isometric measurements of the knee extension strength (∇9.59−13.86%; *d_g_* = 0.36−0.56, small-to-moderate).

Regarding the perceived fatigue assessment, the MST group significantly reduced their FSS scores after the intervention (Δ59.57%; *d_g_* = 5.41, large) compared to the control group. Although part of this gain is maintained at the follow-up assessment (Δ15.10%; *d_g_* = 1.36, large), there is a significant decrease at the follow-up assessment (∇112.08%; *d_g_* = −3.88, large). In addition, the MST group significantly reduced the time invested for completing the TUG test after the intervention (Δ19.69%; *d_g_* = 0.93, large), but a performance decrease is observed when comparing post- vs follow-up assessments (Δ16.93%; *d_g_* = −1.07, large).

## 4. Discussion

Fatigue is considered one of the worst symptoms according to pwMS because of the negative impact it has on their QoL, and the current lack of any medical treatment to reduce it significantly [31,32]. This study aimed to analyse if a maximal resistance-training program, increasing working loads progressively until 90% of RM scores, could improve the perceived-fatigue and functional mobility in pwMS. Our results confirmed the study hypotheses, demonstrating that MST caused an acute significant reduction of perceived-fatigue and enhanced functional mobility in pwMS. However, detraining effects were found in some of the variables after 10 weeks since the post-intervention assessments.

The most important finding of the present study supports the utility of MST to reduce perceived fatigue, showing a great reduction of the FSS scores in the MST group compared to CG, both after training and at the follow-up assessment. These results are in line with those of Kirkergard and colleagues [18] who observed a high reduction in perceived-fatigue (≈64%) in MS participants after an 80% RM training. However, although their improvements in perceived-fatigue levels were slightly higher than those obtained in this study, Kirkergard and colleagues [18] did not present a control group, making it difficult to compare groups and draw conclusions about the impact of the program. The same authors indicated that fatigue reduction could be related to the peripheral inflammatory response that MST program might induce in pwMS [18], reducing pro-inflammatory cytokine levels [19]. Interestingly, it must be noted that the fatigue improvements found in our study were notably greater than those observed in other resistance intervention programs, which oscillated between low-to-moderate effect sizes (0.10‒0.65) [7]. The high fatigue reduction could also be associated with an increase in neural drive [10] and the improvements in lower limb strength after the MST, which is fundamental to reduce the effort needed to perform daily life activities. So, maximizing strength gains after a physical exercise intervention could be the key to counteract the loss of functional mobility and, in consequence, to reduce fatigue in pwMS [12].

Indeed, a positive association between strength gains and functional mobility improvement has been reported in the literature [33]. An improvement in muscle strength allows a person to move more independently and safely in the surrounding environment to accomplish functional activities and increase their participation in the community [32]. Our study orientated the MST program to improve the muscle strength of the lower limbs, and results showed that the training group achieved a 19.89% improvement in the TUG after the intervention. These functional improvements caused by MST might benefit pwMS in moving more efficiently and quickly when performing tasks such as getting up from a chair, turning, or climbing stairs. In this sense, it must be noted that our intervention improves the TUG score to a higher extent than other physical interventions [12], highlighting the potential benefits of MST to improve functionality and the QoL in pwMS [34]. However, the controversy remains; whether this improved functionality is due to the increased strength or the reduced fatigue is still unclear [15].

This study also presented a ten-weeks follow-up stage after the end of the MST program to understand what gains stand up after a period of inactivity, which usually occurs when relapses arise. Results seem to be in line with Dodd and colleagues [35], who found that the benefits on fatigue and muscle strength are quickly getting worse as time goes by once the intervention was finished. Furthermore, it can be observed that not all variables followed the same pattern, that is, the values of isometric and isokinetic strength returned to the initial levels or a little above. These results are in line with Medina-Perez and colleagues’ study [36], where the maximum voluntary isometric contraction of participants returned to pre-training levels after twelve-weeks of detraining. However, the levels of perceived fatigue are striking, although they also worsened, the loss was greater than the strength reduction. Although the reason for the larger worsening that fatigue symptom showed after the detraining period is not clear, our results can be biased by the fact that follow-up measures were performed in September, after the summer period. Authors also think that the larger fatigue worsening highlights the relevance of this symptom for the QoL of this group.

Although our findings seem to highlight the relevance of MST to maximize strength and fatigue improvements, the sample characteristics could have modulated our results in some way. On the one hand, before the intervention, participants in this study were fully sedentary, making them more susceptible to the potential benefits of the training program. On the other hand, in the same way as Dalgas and colleagues [37], our main outcome was the perceived-fatigue, and thus, our group of pwMS presented high and homogeneous FSS scores, most of them categorized as severe fatigue (FSS > 40). All our participants were a specific target of the therapy, which could reveal the real MST effectiveness on fatigue. Therefore, to obtain a more comprehensive knowledge of the efficacy of resistance training programs, future studies should use them with individuals of different degrees of perceived fatigue. In addition, only perceived-fatigue was evaluated in this study, but no information was provided about the improvement in fatigability caused by the MST. Analysing how MST modified some fatigability indexes, it would help to clarify the underlying reasons for our FSS results. Besides, understanding the potential benefits of MST in fatigability could help to optimise training interventions to manage multiple sclerosis-related fatigue. Finally, our perceived-fatigue results could be affected by some psychological and sociological factors like depression [38] or a reduced social activity [39], which, in turn, could be modified by the exercise intervention [40,41]. Futures studies should compare the effect of MST on perceived-fatigue against placebo groups performing a social activity without physical demands.

This study has additional limitations that should be considered to interpret our findings. First, the major one was the small sample size, hindering the result generalization, so larger studies should be performed to confirm the long-term effectiveness of MST on perceived-fatigue symptoms. This sample size also constrained a balanced number of women and men in each group. Second, participants were only relapsing-remitting MS patients presenting a relatively low-to-mild impairment (1.0 < EDSS < 4.5). Hence, the results observed herein cannot be extended to more impaired pwMS or other disease subtypes. Third, no associations between the training gains and the clinical treatments were monitored. Future research would include longer interventions allowing the assessment of both treatments together with other quality of life-related variables such as sleep, mood state, depression, or diet.

## 5. Conclusions

MST seems to be a feasible way to obtain clinically relevant improvements in perceived-fatigue, knee strength and functional mobility. Still, symptom improvements decrease after a 10-week detraining period. Considering the benefits of the MST upon functional mobility and perceived fatigue, this study provides added evidence to those previously indicated in the literature (such as strength gains and lower elevation of body temperature), that is, the feasibility for implementing training programs based on resistance training with high-loads.

## Figures and Tables

**Table 1:** Weekly Training.

	Week	%1RM	Sets	Repetitions	Rest Interval (min)
Pre-test	0	Pre-Intervention measurements			
Conditioning period	1–2	50	2	8–10	3
	3–4	60	2	12–14	3
Maximal strength training period	5–6	75	3	7	5
	1RM measurements for adjusting training loads				
	7–8	80	4	6	5
	9–10	85	4	4	5
	11–12	90	5	4	5
Post-test	13	Post-Intervention measurements			
Follow-up	22	Detraining measurements			

1RM = 1 repetition maximum.

**Table 2:** Training schedule and exercises.

Week	Day	Exercise
1–4	Monday Tuesday Thursday	Dumbbell Shoulder Press Cable Standing Biceps Curl Cable Triceps Pushdown Chest Press Machine Wide-Grip Lat Pulldown Leg Extension Leg Curl Multipower Standing Calf Raises
5–12	1st	Chest Press Machine Barbell Incline Bench Press Leg Curl Leg Extension Multipower Standing Calf Raises
	2nd	Alternate Hammer Curl Cable Triceps Pushdown Front Dumbell Raise Side Lateral Raise
	3rd	Wide-Grip Lat Pulldown Seated Cable Rows Leg Press Thigh Adductor Half Stance Multipower Squat

1st: first training day of the week; 2nd: second training day of the week; 3rd: third training day of the week.

**Table 3:** Participant characteristics.

	MST Group		Control Group		*p*	*d*_g_
Women/Men	9/4		12/1		--	--
Age (years)	45.31	(11.06)	41.31	(9.58)	0.460	0.37
Body mass (kg)	66.02	(15.21)	58.82	(11.31)	0.320	0.52
EDSS (unitless)	2.38	(0.98)	2.81	(1.33)	0.429	−0.36
FSS (unitless)	57.08	(5.92)	52.46	(7.08)	0.084	0.69
TUG (s)	7.06	(1.40)	7.35	(2.10)	0.720	−0.16

Data are presented as mean (standard deviation). MST: Maximum strength training; EDSS: Expanded Disability Status Scale; FSS: Fatigue Severity Scale; TUG: Timed Up and Go Test.

**Table 4:** Strength parameters of the knee extensor and flexor muscles obtained from the isokinetic dynamometer in 60°/s isokinetic and isometric conditions at pre-test, post-test and follow-up test for the maximal strength training (MST) and the control group (CG).

		Pre-Test (1)		Post-Test (2)		Follow-Up (3)		*F*	*p*	ŋp^2^	Dif 1‒2 (%)	*d*_g_ (1‒2)	Dif 1‒3 (%)	*d*_g_ (1‒3)	Dif 2‒3 (%)	*d*_g_ (2‒3)
**QPT_IK_ (Nm/kg)**	**MST**	**2.96**	**(0.72)**	**3.44**	(0.85)	3.11	(0.81)	80.95	<0.001	0.87	Δ16.22	−0.62 **	Δ5.07	−0.20	∇9.59	0.36 **
	CG	3.07	(0.98)	2.60	(1.13)	2.62	(1.05)	7.53	0.018	0.39	∇15.31	0.45 **	∇14.66	0.43 **	Δ0.77	−0.02
HPT_IK_ (Nm/kg)	MST	1.32	(0.36)	1.71	(0.32)	1.62	(0.45)	23.47	<0.001	0.55	Δ29.55	−1.01 **	Δ22.73	−0.78 **	∇5.26	0.26
	CG	1.55	(0.52)	1.55	(0.58)	1.53	(0.49)	0.03	0.879	0.01	0.00	--	∇1.29	0.04	∇1.29	0.03
QPT_IM_ (Nm/kg)	MST	4.26	(1.01)	4.98	(1.16)	4.29	(1.20)	44.17	<0.001	0.79	Δ16.90	−0.67 **	Δ0.70	−0.03	∇13.86	0.56 **
	CG	4.07	(1.54)	3.75	(1.32)	3.71	(1.45)	0.93	0.355	0.07	∇7.86	0.19	∇8.85	0.22	∇1.07	0.03
HPT_IM_ (Nm/kg)	MST	1.71	(0.36)	1.82	(0.31)	1.74	(0.26)	18.37	0.001	0.61	Δ6.43	−0.29 *	Δ1.75	−0.08	∇4.40	0.24
	CG	1.87	(0.75)	1.74	(0.74)	1.82	(0.73)	1.98	0.184	0.14	∇6.95	0.16	∇2.67	0.06	Δ4.60	−0.10
FSS (points)	MST	57.08	(5.92)	22.85	(6.18)	48.46	(9.49)	145.44	<0.001	0.87	Δ59.97	5.41 **	Δ15.10	1.36 *	∇112.08	−3.88 **
	CG	52.46	(7.08)	50.54	(9.71)	51.69	(6.64)	0.79	0.393	0.06	Δ3.66	0.25	Δ1.47	0.10	∇2.28	−0.11
TUG (s)	MST	7.06	(1.40)	5.67	(0.84)	6.63	(1.12)	46.94	<0.001	0.80	Δ19.69	0.93 **	Δ6.09	0.29	∇16.93	−1.07**
	CG	7.35	(2.15)	7.15	(1.99)	8.09	(2.34)	4.10	0.066	0.25	Δ2.72	0.09	∇10.07	−0.32	∇13.15	−0.44

Data are presented as mean (standard deviation). Dif. (%): percentage of difference; Δ: an increase in test performance; ∇: a decrease in test performance; *d_g_:* standardized mean differences between groups calculated with Hedge’s correction; QPT_IK_: Quadriceps Isokinetic Peak Torque normalized by the body mass; HPT_IK_: Hamstring Isokinetic Peak Torque normalized by the body mass; QPT_IM_: Quadriceps Isometric Peak Torque normalized by the body mass; HPT_IM_: Hamstring Isometric Peak Torque normalized by the body mass; FSS: Fatigue Severity Scale; TUG: Timed Up and Go test; MST: maximal strength training; CG: control group. * *p* < 0.05, ** *p* < 0.01.
